# Safety, tolerability, pharmacokinetics, and pharmacodynamics of TG103 injection in participants who are overweight or obese: a randomized, double-blind, placebo-controlled, multiple-dose phase 1b study

**DOI:** 10.1186/s12916-024-03394-z

**Published:** 2024-05-29

**Authors:** Diaozhu Lin, Huisheng Xiao, Kexu Yang, Juan Li, Suiwen Ye, Yanqiong Liu, Shan Jing, Yang Lin, Yuanxun Yang, Lei Huang, Jing Yuan, Ziyan Li, Jinlan Yang, Huanhuan Gao, Ying Xie, Mingtong Xu, Li Yan

**Affiliations:** 1 Department of Endocrinology, Sun Yat-Sen Memorial Hospital, Sun Yat-Sen University, Guangzhou, China; 2 Clinical Pharmacology Centre, Beijing Anzhen Hospital, Capital Medical University, Beijing, China; 3 Phase I Clinical Trials Unit, Nanjing Drum Tower Hospital, The Affiliated Hospital of Nanjing University Medical School, Nanjing, China; 4 Phase I Clinical Trial Center, Sun Yat-Sen Memorial Hospital, Sun Yat-Sen University, Guangzhou, China; 5 CSPC Pharmaceutical Group Co., Ltd, Shijiazhuang, China

**Keywords:** TG103, Overweight, Obese, Safety, Pharmacokinetics, Pharmacodynamics

## Abstract

**Background:**

TG103, a glucagon-like peptide-1 analog, is being investigated as an option for weight management. We aimed to determine the safety, tolerability, pharmacokinetics, and pharmacodynamics of TG103 injection in participants who are overweight or obese without diabetes.

**Methods:**

In this randomized, double-blind, placebo-controlled, multiple-dose phase 1b study, participants aged 18–75 years with a body-mass index (BMI) ≥ 26.0 kg/m^2^ and body weight ≥ 60 kg were enrolled from three centers in China. The study included three cohorts, and in each cohort, eligible participants were randomly assigned (3:1) to one of three once-weekly subcutaneous TG103 groups (15.0, 22.5 and 30.0 mg) or matched placebo, without lifestyle interventions. In each cohort, the doses of TG103 were escalated in 1-week intervals to the desired dose over 1 to 4 weeks. Then participants were treated at the target dose until week 12 and then followed up for 2 weeks. The primary endpoint was safety and tolerability assessed by the incidence and severity of adverse events (AEs) from baseline to the end of the follow-up period. Secondary endpoints included pharmacokinetic and pharmacodynamic profiles of TG103 and the occurrence of anti-drug antibodies to TG103.

**Results:**

A total of 147 participants were screened, and 48 participants were randomly assigned to TG103 (15.0, 22.5 and 30.0 mg groups, *n* = 12 per group) or placebo (*n* = 12). The mean (standard deviation, SD) age of the participants was 33.9 (10.0) years; the mean bodyweight was 81.65 (10.50) kg, and the mean BMI was 29.8 (2.5) kg/m^2^. A total of 466 AEs occurred in 45 of the 48 participants, with 35 (97.2%) in the TG103 group and 10 (83.3%) in the pooled placebo group. Most AEs were grade 1 or 2 in severity, and there were no serious adverse events (SAEs), AEs leading to death, or AEs leading to discontinuation of treatment. The steady-state exposure of TG103 increased with increasing dose and was proportional to C_max,ss_, AUC_ss_, AUC_0-t_ and AUC_0-inf_. The mean values of C_max,ss_ ranged from 951 to 1690 ng/mL, AUC_0-t_ ranged from 150 to 321 μg*h/mL, and AUC_0-inf_ ranged from 159 to 340 μg*h/mL. TG103 had a half-life of 110–116 h, with a median T_max_ of 36–48 h. After treatment for 12 weeks, the mean (SD) values of weight loss from baseline in the TG103 15.0 mg, 22.5 mg and 30.0 mg groups were 5.65 (3.30) kg, 5.35 (3.39) kg and 5.13 (2.56) kg, respectively, and that in the placebo group was 1.37 (2.13) kg. The least square mean percent weight loss from baseline to D85 in all the TG103 groups was more than 5% with *p* < 0.05 for all comparisons with placebo.

**Conclusions:**

In this trial, all three doses of once-weekly TG103 were well tolerated with an acceptable safety profile. TG103 demonstrated preliminary 12-week body weight loss without lifestyle interventions, thus showing great potential for the treatment of overweight and obesity.

**Trial registration:**

ClinicalTrials.gov, NCT04855292. Registered on April 22, 2021.

**Supplementary Information:**

The online version contains supplementary material available at 10.1186/s12916-024-03394-z.

## Background

Overweight and obesity are defined by the World Health Organization (WHO) as abnormal or excessive fat accumulation that may present a health risk, which is an increasing global public health issue [1]. The WHO has identified obesity as the most prevalent chronic disease worldwide and estimated that globally, there are more than 650 million people with obesity [2]. China is one of the countries with the fastest rate of increasing obesity prevalence in the world [3]. Therefore, obesity and its associated health problems may also have a significant economic impact on the health care system [4].

Overweight and obese populations are at major risk for a range of comorbidities, including cardiovascular disease, gastrointestinal disease, type-2 diabetes, joint and muscle diseases, respiratory problems, and psychological problems that can significantly impact their daily lives and increase the risk of death [5, 6]. The chance of developing diabetes, heart disease, and other weight-related health risks increases with increasing body mass index (BMI) [7]. There is also strong evidence that at any given BMI, these health risks are markedly higher in some ethnic groups than in others. It appears that Asians experience higher risks of hypertension and cardiovascular disease at lower levels of BMI compared to other racial groups [8].

The development of obesity is mainly caused by complex interactions between genetic, metabolic, neuroendocrine, behavioral, and environmental factors involved in the regulation of energy balance and fat storage [9]. Lifestyle modification is the recommended foundation of treatment for individuals with overweight or obesity [10]. As lifestyle and behavioral interventions result in moderate weight loss, which is often regained, obesity treatment strategies should be escalated by the addition of pharmacological and/or surgical interventions if necessary [11]. Surgical treatments include liposuction, lipectomy and various procedures to reduce food absorption, but they may cause complications such as malnutrition, anemia and narrowing of the digestive tract and thus require strict indications [12]. Meanwhile, surgical interventions have high requirements for indications and are incapable of satisfying the treatment needs of all overweight and obese populations. Thus, pharmacological interventions may be used in the management of obesity as an adjunct to lifestyle modification as the second-line treatment for obesity [13]. Pharmacological development for obesity has been hampered by concerns over safety and a paucity of substantial efficacy. Historically, there has been a collection of anti-obesity medication failures that have occurred after regulatory approval, due to adverse cardiovascular effects, increased suicidal risk or enhanced likelihood of drug dependence and abuse [11]. However, the use of glucagon-like peptide 1 receptor agonists (GLP-1 RAs) is promoting the belief that drug-based management of obesity may be possible for long-term treatment with suitable tolerability and safety. GLP-1, an enteric-derived hormone secreted by the human intestine, has shown great potential in both diabetes and obesity treatment. GLP-1 can reduce body weight in various ways, including inhibiting gastrointestinal motility and gastric juice secretion, suppressing appetite, delaying gastric emptying and acting on the central nervous system (especially the hypothalamus) to produce a feeling of satiety in humans [14]. Several GLP-1 RAs have been approved for the treatment of obesity. Liraglutide was initially used to treat type 2 diabetes and was later approved by the U.S. Food and Drug Administration (FDA) in 2014 for the treatment of obesity due to its demonstrated effects on weight loss [15]. Semaglutide is a longer-acting GLP-1 RA that was also approved by the FDA for weight management in the United States on 4 June 2021 [16, 17]. However, in China, orlistat is the only approved agent for this setting. No GLP-1 RAs are currently approved for the treatment of overweight or obesity in China. Therefore, there are unmet medical needs for this population in China.

TG103 is a recombinant protein that uses Fc-fusion protein technology to link human GLP-1 and Fc fragments via IgD, which could avoid lysosomal degradation and reduce renal clearance. Preclinical pharmacological tests have shown an affinity for the GLP-1 receptor and a long half-life, making it possible to provide patients with weekly injections of the GLP-1 analog in clinical treatment. The results of a previous single ascending dose (SAD) study of TG103 injection in a healthy population showed good tolerability and safety of TG103 [18]. Nonclinical rodent studies have shown that TG103 reduces body weight and food intake, consistent with other GLP-1 RAs that reduce body weight primarily through appetite suppression (data not shown).

This study aimed to investigate the safety, tolerability, pharmacokinetics, and pharmacodynamics of once-weekly subcutaneous TG103 injection (target doses 15.0, 22.5, and 30.0 mg) versus placebo in nondiabetic participants with overweight or obesity.

## Methods

### Study design

This was a randomized, double-blind, placebo-controlled, multiple-dose clinical trial to evaluate the safety, tolerability, pharmacokinetic (PK) and pharmacodynamic (PD) characteristics of TG103 in participants who are overweight or obese without diabetes. The trial included 3 periods: an approximately 4-week screening period, a 12-week dosing period and a 2-week follow-up period. Eligible participants were enrolled into three paralleled dose cohorts (15.0, 22.5 and 30.0 mg) with 16 participants in each cohort. Within each cohort, participants were randomized in a 3:1 ratio to receive TG103 injection or placebo subcutaneously (SC) once a week (QW) for 12 weeks. Each cohort was started at a low dose of 7.5 mg and gradually uptitrated at weekly intervals until the target dose of each cohort was reached.

The trial was conducted at three centers in China and was sponsored by CSPC Baike (Shandong) Biopharmaceutical Co., Ltd. The trial was reviewed and approved by the institutional review board or ethics committee of each participating center and was conducted in accordance with the principles of the Declaration of Helsinki. All the participants provided written informed consent before study initiation. The trial was registered at clinicaltrials.gov (NCT04855292).

### Participants

Eligible participants were aged between 18 and 75 years (inclusive) with a BMI ≥ 26.0 kg/m^2^. Participants should weigh 60 kg or more and remain stable in weight (self-reported weight change of less than 5% within 3 months). The criteria for inclusion also included fasting blood glucose of 3.9–7.0 mmol/L (exclusive) and HbA1c < 6.5%. Key exclusion criteria included a confirmed diagnosis of type-1 or type-2 diabetes mellitus, and the presence of secondary obesity induced by metabolic disorders (e.g., Cushing’s syndrome, hypothyroidism, etc.) or drug treatment (e.g., glucocorticoids, tricyclic antidepressants, atypical antipsychotics, etc.). Participants who had a history of allergy to any GLP-1 analogs, or who have had previous severe allergic reactions to food or drugs, etc., were also excluded from the trial.

### Randomization and blinding

Eligible participants were randomized within each cohort in a 3:1 ratio using a block scheme via an interactive web-response system. The randomization schedule was prepared by the sponsor. All the study medications were in sealed packages with similar appearances. Participants, investigators, study site staff and sponsor representatives were blinded to the assigned study treatment.

### Procedures

Eligible participants were randomized to receive either TG103 injection or placebo in three paralleled dose cohorts: cohort A, 15.0 mg dose group (TG103: placebo = 12:4); cohort B, 22.5 mg dose group (TG103: placebo = 12:4); and cohort C, 30.0 mg dose group (TG103: placebo = 12:4). Participants in each cohort were started at a low dose of 7.5 mg and gradually increased over 1 to 4 weeks to the target dose, which was administered once a week for 12 weeks.

Blood samples for PK were collected after the first target dose and after the last target dose (12th dose) to measure TG103 concentrations. Blood samples of 3.5 mL for PK analysis were collected from participants at prespecified time points (Additional file 1: Table S1) to assess the serum concentrations of TG103. Predose blood samples of 3.5 mL for immunogenicity tests were collected on Days 1, 15, 29, and 57, and Day 99 (21 ± 2 days after the last dose). Samples were tested for anti-drug antibodies (ADA).

### Outcomes and assessments

The primary endpoint was safety and tolerability assessed by the incidence and severity of adverse events (AEs) from baseline to the end of the follow-up period. Safety assessments included physical examination, vital signs, laboratory examination, electrocardiogram (ECG) and AEs. Secondary endpoints included PK and PD profiles of TG103 and occurrence of TG103 ADA. PD endpoints were comprised the proportion of participants with a baseline weight loss of more than 5%, and change from baseline to end of treatment (defined as 1 week after the last dose) in weight, waistline, waist-hip ratio, blood pressure (systolic blood pressure and diastolic blood pressure) and blood lipase levels.

Serum concentrations of TG103 was detected using a double-antibody sandwich assay. The method was established and validated by central lab. The PK parameters of TG103 were derived using noncompartmental methods and comprised steady-state maximum concentration (C_max,ss_), time to maximum concentration (T_max_), area under the concentration–time curve from time zero to time of the last measurable concentration (AUC_0-t_), area under the concentration–time curve from time zero to infinity (AUC_0-inf_), area under the concentration–time curve during a dosing interval at steady state (AUC_ss_), average steady-state concentration during a dosing interval (C_avg,ss_), steady-state minimum concentration (C_min,ss_), steady-state apparent total body clearance (CL_ss_/F), apparent total volume of distribution (V_z_/F), and terminal half-life (t_1/2_). The immunogenicity and presence of ADA were determined by a validated immunoassay on an electrochemiluminescence (ECL) platform to detect ADA against TG103.

### Statistical analysis

The sample size was not based on a formal statistical power calculation. The sample size of 16 participants per group was empirically determined to provide preliminary safety, tolerability, PK and PD data. No formal hypothesis testing was planned. The dose selection for this study was based on completed animal trials and the results of the first SAD trials in healthy participants conducted in Germany and China [18].

Baseline characteristics were summarized by descriptive statistics, using number and proportion for categorical data and mean (standard deviation, SD), mean (standard error, SE) or median (range) for continuous data (full analysis set, FAS). Safety endpoints were analyzed in all participants who were exposed to at least one dose of the trial product (safety analysis set, SS) and summarized by descriptive statistics. PK endpoints were analyzed in all participants randomly assigned to treatment who received TG103 with at least one measurable concentration (pharmacokinetic concentration analysis set, PKCS) or with at least one valid PK parameter (pharmacokinetic parameter analysis set, PKPS). PD endpoints were analyzed in all participants who randomly received a trial product with at least one efficacy result (pharmacodynamics analysis set, PDS). Immunogenicity analyses were performed in the safety population (SS).

For analyses of the primary endpoint of safety and the secondary endpoints of pharmacodynamics, participants who were given placebo were pooled across groups. Safety analyses were performed using descriptive statistics using National Cancer Institute (NCI) Common Terminology Criteria for Adverse Events (CTCAE) version 4.03. All analyses were performed with SAS 9.4 software. Non-compartmental analysis for PK parameters was performed with Phoenix WinNonlin v8.2. The power function model was used to evaluate the linear relationship between the exposure (C_max,ss_, AUC_ss_, AUC_0-t_ and AUC_0-inf_) and dose: PK parameter: ln (y) = β_0_ + β_1_* ln (Dose), where 90% CI of slope β_1_ point estimate including 1 indicates dose-proportional increase.

Based on the PDS, an analysis of covariance (ANCOVA) was conducted to evaluate the effect of each dose group of TG103 on body weight after 12 weeks of treatment, using the dose group as a fixed effect and the baseline body weight as a covariate. Logistic regression analysis based on PDS, with dose group as a fixed effect and baseline body weight as a covariate, was performed to evaluate the difference between the effect of each dose group of TG103 on body weight and that of placebo, using the proportion of participants with a ≥ 5% reduction in body weight from baseline after 12 weeks of treatment.

## Results

### Participants

**Fig. 1:**
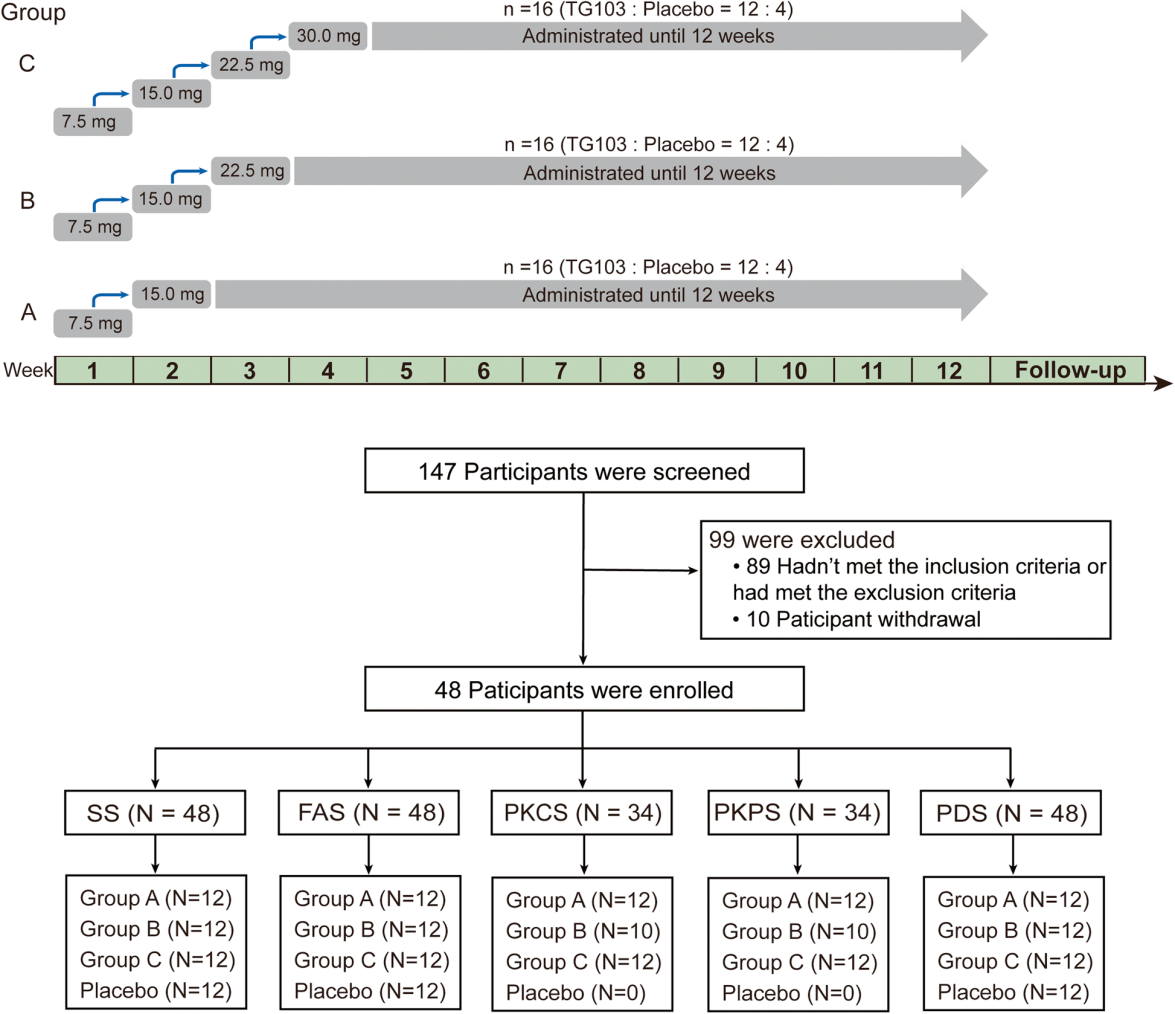
Study design and overall participant distribution

Between July 25, 2018, and Dec 17, 2019, a total of 147 participants were screened for eligibility, of whom 48 were enrolled. In each group, 16 participants were randomized, with 12 assigned to TG103 and 4 assigned to placebo (Fig. 1). Five participants withdrew from the study early, of whom 3 withdrew consent voluntarily, 1 was lost to follow-up, and 1 quit for other reasons. The remaining 43 participants (89.6%) completed the trial. All randomized participants (*n* = 48) were exposed to treatment and were included in the FAS, SS and PDS. Except for 2 participants in the 22.5 mg group who reported major deviation, which may affect the PK analysis, all the other participants receiving TG103 (*n* = 34) were included in the PKCS and PKPS. Thirty (62.5%) of 48 participants were males. The mean (SD) age of the participants was 33.9 (10.0) years; the mean bodyweight was 81.65 (10.50) kg, the mean BMI was 29.8 (2.5) kg/m^2^, and the mean waist circumference was 97.98 (7.52) cm. Baseline characteristics were generally similar across treatment groups (Table 1).

**Table 1:** Baseline demographics characteristics

**Characteristics**	**TG103, 15.0 mg** **(*N*= 12)**	**TG103, 22.5 mg** **(*N = 12)***	**TG103, 30.0 mg** **(*N = 12)***	**Pooled placebo** **(*N = 12)***	All **(*N = 48)***
Age, years	33.3 (8.0)	28.8 (7.6)	38.8 (11.8)	34.5 (10.6)	33.9 (10.0)
Sex					
Female	4 (33.3%)	3 (25.0%)	6 (50.0%)	5 (41.7%)	18 (37.5%)
Male	8 (66.7%)	9 (75.0%)	6 (50.0%)	7 (58.3%)	30 (62.5%)
Bodyweight, kg	79.68 (6.86)	85.77 (12.97)	81.03 (10.43)	80.07 (10.96)	81.65 (10.50)
BMI, kg/m^2^	29.0 (2.4)	30.4 (3.1)	30.3 (2.6)	29.4 (1.9)	29.8 (2.5)
Waist circumference, cm	98.38 (5.30)	100.38 (8.99)	97.36 (7.18)	95.78 (8.62)	97.98 (7.52)
Blood pressure, mmHg					
Systolic	115.9 (14.9)	119.9 (14.9)	124.1 (16.5)	115.5 (10.2)	118.9 (14.1)
Diastolic	75.3 (9.9)	70.3 (10.2)	73.3 (11.6)	71.5 (9.7)	72.6 (10.4)
Lipids, mmol/L					
Total cholesterol	4.569 (0.889)	4.561 (0.863)	5.016 (0.871)	4.767 (0.862)	4.728 (0.871)
LDL-C	2.843 (0.677)	2.876 (0.776)	3.133 (0.694)	2.913 (0.797)	2.941 (0.736)
Triglycerides	1.735 (0.515)	1.369 (0.745)	1.590 (0.579)	1.388 (0.603)	1.521 (0.611)

Data are mean (SD) or *n* (%), unless otherwise specified. Data for participants receiving treatment with placebo were pooled across groups

*BMI* body-mass index, *LDL-C* low density lipoprotein cholesterol

### Safety

A total of 466 AEs occurred in 45 of the 48 participants, with 35 (97.2%) in the TG103 group and 10 (83.3%) in the pooled placebo group. Except for 2 participants in the TG103 22.5 mg dose group and 1 participant in the placebo group who had grade 3 AEs deemed to be related to treatment by the investigator, all other participants had grade 1 or 2 AEs. There were no SAEs, no AEs leading to death, and no AEs leading to discontinuation of treatment.

The AEs occurring in at least 20% of participants in the TG103 group were decreased appetite (58.3%), nausea (55.6%), diarrhea (52.8%), injection-site reaction (47.2%), hypertriglyceridemia (33.3%), vomiting (33.3%) and hyperuricemia (22.2%). The AEs occurring in at least 20% of participants in the pooled placebo group were hypertriglyceridemia (41.7%), decreased appetite (25.0%), and hyperuricemia (25.0%).

The most commonly reported AEs were gastrointestinal disorders, with 58.3% in the 15.0 mg group, 91.7% in the 22.5 mg group, 83.3% in the 30.0 mg group and 16.7% in the placebo group. Among the gastrointestinal disorders, the incidence of vomiting (25.0% in the 15.0 mg group, 33.3% in the 22.5 mg group, 41.7% in the 30.0 mg group, 0 in the placebo group), diarrhea (33.3% in the 15.0 mg group, 50.0% in the 22.5 mg group, 75.0% in the 30.0 mg group, 8.3% in the placebo group), and abdominal distension (0 in the 15.0 mg group, 25.0% in the 22.5 mg group, 33.3% in the 30.0 mg group, 8.3% in the placebo group), increased with the increasing dose to some extent. The injection-site reaction also showed a dose-dependent increase of 33.3% in the 15.0 mg group, 50.0% in the 22.5 mg group, 58.3% in the 30.0 mg group and 8.3% in the placebo group.

Decreased appetite was reported as an AE more frequently with higher doses of TG103 with 50.0% in the 15.0 mg group, 50.0% in the 22.5 mg group, 75.0% in the 30.0 mg group and 25.0% in the placebo group. A summary of treatment-emergent AEs is presented in Table 2.

**Table 2:** Treatment-emergent adverse events

	**TG103, 15.0 mg** **(*N* = 12)**	**TG103, 22.5 mg** **(*N* = 12)**	**TG103, 30.0 mg** **(*N* = 12)**	**TG103 total** **(*N* = 36)**	**Pooled placebo** **(*N* = 12)**
Treatment-emergent adverse events	11 (91.7%)	12 (100.0%)	12 (100.0%)	35 (97.2%)	10 (83.3%)
Treatment-related	9 (75.0%)	11 (91.7%)	12 (100.0%)	32 (88.9%)	4 (33.3%)
Grade ≥ 3 adverse events	0	2 (16.7%)	0	2 (5.6%)	1 (8.3%)
Treatment-related	0	2 (16.7%)	0	2 (5.6%)	1 (8.3%)
Adverse events of gastrointestinal disorders	7 (58.3%)	11 (91.7%)	10 (83.3%)	28 (77.8%)	2 (16.7%)
Adverse events by preferred term^a^					
Decreased appetite	6 (50.0%)	6 (50.0%)	9 (75.0%)	21 (58.3%)	3 (25.0%)
Nausea	4 (33.3%)	8 (66.7%)	8 (66.7%)	20 (55.6%)	0
Diarrhea	4 (33.3%)	6 (50.0%)	9 (75.0%)	19 (52.8%)	1 (8.3%)
Injection-site reaction	4 (33.3%)	6 (50.0%)	7 (58.3%)	17 (47.2%)	1 (8.3%)
Hypertriglyceridemia	3 (25.0%)	4 (33.3%)	5 (41.7%)	12 (33.3%)	5 (41.7%)
Vomiting	3 (25.0%)	4 (33.3%)	5 (41.7%)	12 (33.3%)	0
Hyperuricemia	4 (33.3%)	1 (8.3%)	3 (25.0%)	8 (22.2%)	3 (25.0%)
Abdominal distension	0	3 (25.0%)	4 (33.3%)	7 (19.4%)	1 (8.3%)
Electrocardiogram T wave abnormal	3 (25.0%)	2 (16.7%)	0	5 (13.9%)	0
Hypercholesterolemia	3 (25.0%)	0	1 (8.3%)	4 (11.1%)	2 (16.7%)

Data are *n* (%), where n is participants with one or more adverse events. Data for participants receiving treatment with placebo were pooled across cohorts

^a^Adverse events occurring in at least 20% of participants in any group

### Pharmacokinetics

**Fig. 2:**
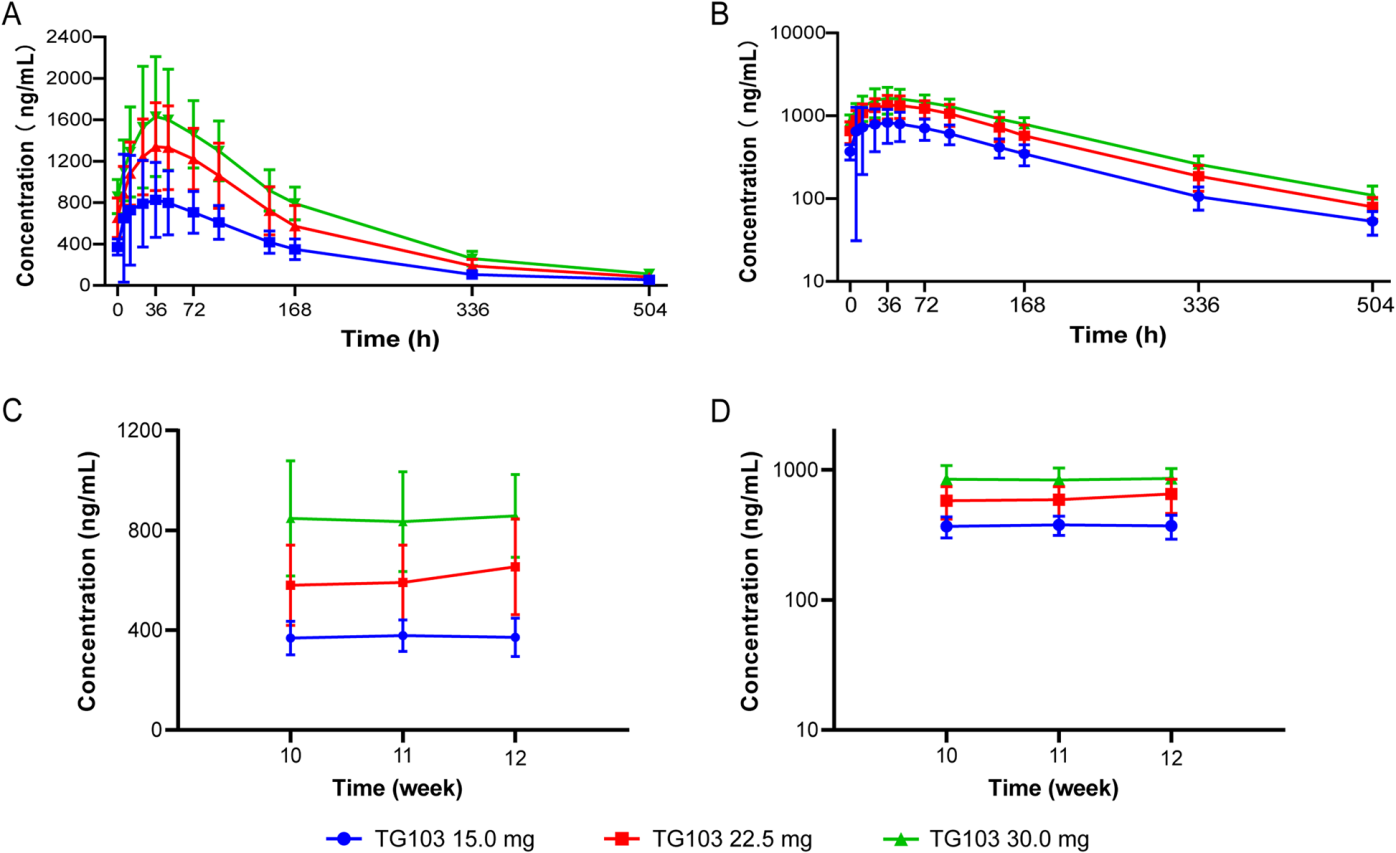
Pharmacokinetic profile of TG103. Serum concentration–time curves of TG103 after the last target dose: (**A**) Linear plots and (**B**) Semilogarithmic plots. The blood concentration of TG103 before the 10th, 11th and 12th doses in each dose group: (**C**) Linear plots and (**D**) Semilogarithmic plots. Error bars represent standard deviation

Following subcutaneous administration, TG103 demonstrated slow absorption with C_max_ ranging from 5.85 to 96.00 h, and the median T_max_ was 36.02, 35.82 and 48.03 h for doses of 15.0, 22.5 and 30.0 mg after the last target dose, respectively. After the last target dose, the mean values of C_max,ss_ were 951, 1410 and 1690 ng/mL, respectively; AUC_0-t_ ranged from 150 to 321 μg*h/mL, and AUC_0-inf_ ranged from 159 to 340 μg*h/mL. The serum concentration–time curves of TG103 after the last target dose of each group are shown in Fig. 2A&B. A summary of the PK parameters of TG103 after the last target dose is shown in Table 3. The steady-state exposure to TG103 increased with increasing dose and was proportional to C_max,ss_, AUC_ss_, AUC_0-t_ and AUC_0-inf_. The β1 point estimates (90% confidence interval, [CI]) of C_max,ss_, AUC_ss_, AUC_0-t_ and AUC_0-inf_ were 0.972 (0.329, 1.616), 1.085 (0.604, 1.566), 1.133 (0.683, 1.583) and 1.129 (0.683, 1.574), respectively. The blood concentration of TG103 stabilized before the 10th, 11th and 12th doses in each dose group, which demonstrated that TG103 had reached a steady state by the 10th dose (Fig. 2C&D). A summary of the PK parameters of TG103 after the first target dose is shown in Additional file 1: Table S2 and the serum concentration–time curves of TG103 after the first target dose are shown in Additional file 1: Fig. S1.

**Table 3:** Summary of pharmacokinetic parameters of TG103 after the last target dose

	**15.0 mg** **(*N* = 11)**	**22.5 mg** **(*N* = 10)**	**30.0 mg** **(*N* = 10)**
T_max_ (h)	36.02 (5.85, 96.00)	35.82 (23.95, 72.02)	48.03 (35.85, 95.83)
C_max,ss_ (ng/mL)	951 ± 573	1410 ± 413	1690 ± 507
C_min,ss_(ng/mL)	323 ± 65.6	559 ± 174	766 ± 154
C_avg,ss_(ng/mL)	612 ± 194	1020 ± 280	1260 ± 280
AUC_0-t_ (μg*h/mL)	150 ± 41.7	249 ± 70.3	321 ± 60.7
AUC_0-inf_ (μg*h/mL)	159 ± 43.6	261 ± 73.5	340 ± 63.7
AUC_ss_(μg*h/mL)	103 ± 32.6	171 ± 46.9	211 ± 46.8
t_1/2_ (h)	116 ± 10.8	110 ± 11.9	116 ± 9.34
V_z_/F (L)	26.9 ± 9.25	23.0 ± 9.47	25.2 ± 6.59
CL_ss_/F (L/h)	0.16 ± 0.05	0.14 ± 0.06	0.15 ± 0.04

Data are described by mean ± SD except for T_max_ showed as median (min, max)

*AUC*_*0-t*_ area under the concentration‐time curve from time zero to time of the last measurable concentration, *AUC*_*0-inf*_ area under the concentration‐time curve from time zero to infinity, *AUC*_*ss*_ area under the concentration–time curve during a dosing interval at steady state, *C*_*max,ss*_ steady-state maximum concentration, *C*_*min,ss*_ steady-state minimum concentration, *C*_*avg,ss*_ average steady-state concentration during a dosing interval, *CL*_*ss*_*/F* steady-state apparent total body clearance, *t*_*1/2*_ terminal half-life, *T*_*max*_ time to maximum concentration, *SD* standard deviation, *V*_*z*_*/F* apparent total volume of distribution

### Immunogenicity

The incidence of antibodies to TG103 was 22.2% (8/36), with 5 participants (41.7%) in the TG103 15.0 mg group, 1 participant (8.3%) in the 22.5 mg group and 2 participants (16.7%) in the 30.0 mg group testing positive for immunogenicity. All participants in the placebo group tested negative for immunogenicity during the test.

The results of the exploratory analysis of the effect of immunogenicity on PK showed that exposure levels were lower in immunogenic-positive participants in the 15.0 mg group (C_max,ss_ of 669 ng/mL and AUC_0-t_ of 134 μg*h/mL) than in negative participants (C_max,ss_ of 1190 ng/mL and AUC_0-t_ of 163 μg*h/mL). Similar results were also observed for AUC_ss_ and AUC_0-inf_ in immunogenic-positive participants in the 15.0 mg group. Due to the low number of immunogenic-positive participants in the 22.5 mg and 30.0 mg groups, no further analysis was performed.

### Pharmacodynamics

**Fig. 3:**
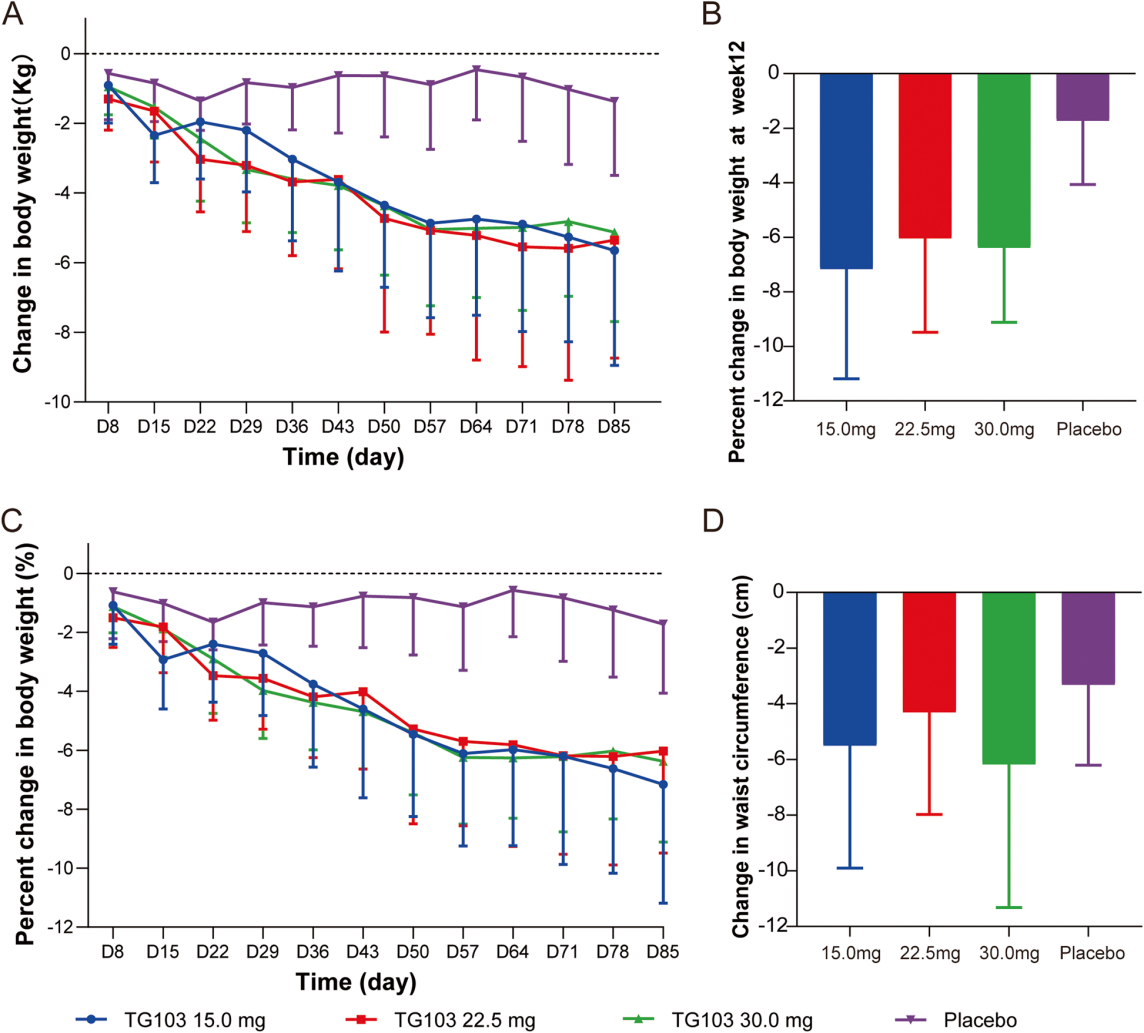
Change from baseline in body weight and waist circumference. **A** Change from baseline in body weight over time. **B** Change from baseline in body weight at week 12. **C** Percentage change from baseline in body weight over time. **D** Change from baseline in waist circumference over time. Data are plotted as the mean (SD)

Based on the PDS, the mean weight change from baseline by day and the mean percent weight loss from baseline by day for each dose group are shown in Fig. 3A&C. The results of the change in efficacy outcomes from baseline to week 12 are presented in Table 4. The mean (SD) values of D85 weight loss from baseline in the TG103 15.0 mg, 22.5 mg and 30.0 mg groups were 5.65 (3.30) kg, 5.35 (3.39) kg and 5.13 (2.56) kg, respectively, and the mean (SD) values of D85 weight loss from baseline in the placebo group were 1.37 (2.13) kg (Fig. 3B).

**Table 4:** Change in efficacy outcomes from baseline to week 12

**Characteristics**	**TG103, 15.0 mg** **(*N* = 12)**	**TG103, 22.5 mg** **(***N*** = 12)**	**TG103, 30.0 mg** **(*N* = 12)**	**Pooled placebo** **(*N* = 12)**
Body weight, Kg				
Mean (SD)	-5.65 (3.30)	-5.35 (3.39)	-5.13 (2.56)	-1.37 (2.13)
Range (Minimum, Maximum)	-13.0, -1.1	-11.0, 0	-10.2, -2.5	-5.9, 2.9
Body weight, %				
LSM (SE)	-7.24 (0.97)	-5.79 (1.05)	-6.47 (1.02)	-1.76 (0.93)
LSM difference between TG103 and placebo (95% CI)	-5.48 (-8.77, -2.19)	-4.03 (-7.51, -0.55)	-4.71 (-8.08, -1.33)	
* p* value	0.0006	0.0195	0.0043	
Weight loss ≥ 5% at week 12, No. (%)	8 (66.7%)	5 (41.7%)	6 (50.0%)	1 (8.3%)
* p* value	0.0057	0.0785	0.0192	
Waist circumference, cm	-5.50 (4.40)	-4.30 (3.68)	-6.17 (5.15)	-3.31 (2.90)
Waist-to-hip ratio	-0.022 (0.037)	-0.019 (0.040)	-0.013 (0.030)	-0.015 (0.019)
Blood pressure, mmHg				
Systolic	-0.3 (11.8)	-5.6 (10.5)	-16.1 (7.9)	2.5 (12.7)
Diastolic	-2.6 (6.5)	-0.7 (10.8)	-4.1 (4.3)	2.8 (8.4)

Data are presented as mean (SD) change from baseline to week 12, unless otherwise specified. *LSM* least square mean

The least square mean (SE) percent weight loss from baseline to D85 in the TG103 15.0 mg, 22.5 mg, and 30.0 mg groups were -7.24 (0.97)%, -5.79 (1.05)%, and -6.47 (1.02)%, respectively, and that of the placebo group was -1.76 (0.93)%. There was also a significant difference in each dose group compared to the placebo group (*p* < 0.05 for all comparisons). The least square mean differences between each dose group and placebo (TG103 group—placebo group) were -5.48% (95% CI, -8.77, -2.19), -4.03% (95% CI, -7.51, -0.55), and -4.71% (95% CI, -8.08, -1.33), respectively. The upper 95% CIs for the least square mean differences were less than 0, and the p values were all less than 0.05. Therefore, the weight reduction in each TG103 dose group after 12 weeks of treatment was better than that in the placebo group.

The odds ratios (95% CI) of the proportion of participants in the TG103 15.0, 22.5 and 30.0 mg groups who showed a ≥ 5% reduction in body weight from baseline over that in the placebo group were 33.73 (95% CI, 2.78, 408.63), 8.93 (95% CI, 0.78, 102.43), and 19.14 (95% CI, 1.62, 226.56), respectively. Except for the TG103 22.5 mg group, the 95% CIs of the odds ratio in both the TG103 15.0 and 30.0 mg groups did not contain 1, and the p values were less than 0.05; therefore, the proportion of participants with a ≥ 5% reduction in body weight from baseline was significantly higher in the TG103 15.0 mg and 30.0 mg groups than in the placebo group.

After 12 weeks of treatment, waist circumference (Fig. 3D) and waist-to-hip ratio decreased in each group compared to baseline, but there was no significant difference between each group and the placebo group. The systolic blood pressure decreased the most in the 30.0 mg group, followed by 22.5 mg, and decreased less in the 15.0 mg and placebo groups. The diastolic blood pressure in all TG103 treatment groups showed some reduction from the baseline from the placebo group.

## Discussion

This was a randomized, double-blind, placebo-controlled, multiple-dose clinical trial to evaluate the safety, tolerability, PK and PD characteristics of different doses of once-weekly TG103 in overweight or obese participants without diabetes mellitus. Overall, in this phase 1b trial, treatment with TG103 15.0–30.0 mg for 12 weeks was generally well tolerated in participants with overweight or obesity, showing preliminary efficacy in weight loss.

In our previous SAD study, a single ascending dose of TG103 (3.0–22.5 mg) was evaluated in healthy Chinese participants. Here, we added a higher dose of 30.0 mg in this study. That decision was based on a PopPK analysis that was performed on concentration data of TG103 in healthy Chinese subjects. Body weight and sex as covariates were the main factors affecting the clearance and volume of distribution of TG103 in vivo, and body weight had a greater effect on the volume of distribution and clearance. It was shown that the higher the body weight, the higher the clearance, and therefore the in vivo exposure was relatively low in the obese population when given the same dose (data not shown). Meanwhile, the additional 30.0 mg dose group in this trial is also within the safe range, considering the maximum recommended starting dose (MRSD) of 58 mg based on the preclinical crab monkey study.

The overall safety and tolerability of the trial was good. No participants withdrew because of AEs, while four withdrew for personal reasons and one was lost to follow-up. In this trial, most AEs were CTCAE grade 1 or 2 in severity. Three participants experienced grade 3 AE, with 2 participants in the 22.5 mg group (hypertriglyceridemia and blood creatine phosphokinase increased) and 1 in the placebo group (blood creatine phosphokinase increased). The occurrence of gastrointestinal disorders was expected because of the known effect of GLP-1 analogs on gastrointestinal motility and was in line with previous studies [19, 20]. The incidence of gastrointestinal-related AEs, such as decreased appetite, vomiting, diarrhea, and abdominal distension, showed a dose-dependent increase to some extent. Similar to other previous studies, more gastrointestinal-related AEs were observed in the higher-dose TG103 group, while all AEs were mild to moderate and resolved without any intervention. In this study, the use of dose titration has been observed in clinical practice to mitigate adverse gastrointestinal reactions and improve patient clinical tolerance of GLP-1 agonists. Decreased appetite is classified as a nutritional and metabolic disease according to the system organ class of AEs; however, it is commonly considered to be associated with delayed gastric emptying and centrally mediated effects. Since GLP-1 has a role in suppressing appetite, the participants receiving GLP-1 agonists usually experience decreased appetite, and we also observed a high incidence of decreased appetite (58.3%) in the TG103 group. The injection-site reaction also showed a dose-dependent increase. Increased heart rate has been reported after some GLP-1 RAs administration in several studies. Our preclinical evaluation in cynomolgus monkeys showed that TG103 had no effect on heart rate, and this finding has also been confirmed in previous Phase I clinical trials conducted in both German and Chinese healthy subjects. In this study, there was one case of increased heart rate reported in the TG103 30 mg group. The AE was graded as 1 according to CTCAE 5.0 and judged by the investigator to be unrelated to the drug.

Based on the results of a SAD study of a single dose of TG103, in which the mean half-life of different doses of TG103 (3.0, 7.5, 15.0 and 22.5 mg) was 147.16 to 184.72 h, a once-weekly dose interval was used in this study. This dosing interval was also similar to other weight reduction studies of GLP-1 receptor agonists, such as semaglutide [20]. In this study, we reported the PK profile of TG103 after multiple doses. Before the 10th dose, TG103 reached a steady state. The mean serum concentration of TG103 after subcutaneous administration of 15.0 to 30.0 mg showed a dose-dependent increase, with a similar course of change in TG103 concentrations over time in all dose groups. The T_max_ values at different doses were similar to those reported in the SAD study. The t_1/2_ of TG103 in this study was between the values reported in the single-dose study of healthy Chinese and German populations [18]. The exposure of the TG103 15.0 mg and 22.5 mg groups after the last target dose was higher than that after the first target dose. To a certain extent, this demonstrated that there was some accumulation of TG103 after multiple doses. However, since the first target dose was not the first dose administered to the participants, the accumulation ratio of TG103 was not calculated in this study.

This study initially explored the effect of immunogenicity on the PK profile, and the preliminary results showed that participants with positive immunogenicity had lower levels of drug exposure than those with negative immunogenicity in the 15.0 mg group. However, due to the limited number of immunogenic-positive participants in each group of this study, there is insufficient evidence to characterize the effect of ADA on the PK of TG103. Further studies with larger sample sizes are needed to evaluate the impact of ADA on clearance of TG103.

According to the preclinical animal pharmacodynamic model, TG103 at doses of 2.5, 7.5, and 22.5 mg/kg all significantly reduced body weight and body weight growth rate in obese mice, with a dose correlation between 2.5 mg/kg and 7.5 mg/kg, which converted to approximately 12.0 mg and 36.0 mg in humans. Meanwhile, based on the safety results of our previous phase I study, therefore, three dose levels between 12.0 to 36.0 mg (15.0, 22.5 and 30.0 mg) were selected for the weight loss study in this trial. In this study, we preliminarily assessed the weight loss efficacy of TG103 at three dose levels in participants with overweight and obesity without diabetes. Mean weight reductions were seen in all the dose groups of TG103 without lifestyle interventions, showing obvious differences compared to the placebo group. Weight loss of at least 5% has been associated with improvements in cardiovascular risk factors, prevention of type-2 diabetes, and improvements in obesity-associated complications [20]. In this study, there were several participants in each dose group achieved a weight loss of > 5%. There was also a reduction in other secondary endpoints of efficacy, such as waist circumference, waist-to-hip ratio, blood pressure, etc., to some extent. However, there was no significant difference between the TG103 treatment group and the placebo group. The treatment period of this study was 12 weeks, which also included the incremental process of different dose groups; therefore, the treatment period of different dose groups at the target dose was different. Meanwhile, the overall treatment period was also shorter compared to the other phase II or phase III studies in which weight loss was chosen as the primary endpoint [21–23]. Therefore, only preliminary efficacy was observed in this trial. Furthermore, TG103 was observed to have a similar effect on weight loss in the three dose groups in this trial, which may be related to factors such as the short treatment time, small sample size, and differences in diet and exercise among participants in different dose groups in this study. It’s necessary to be further verified in studies with a larger sample size and long-term treatment to more adequately assess the efficacy and dose–effect relationship of TG103.

There were some limitations in this trial. This trial had a relatively short duration and a small sample size. The short duration of the trial and the short time that participants were treated at the final target dose might have restricted treatment efficacy and safety assessments. The sample size might limit further analysis of the effect of immunogenicity on the PK profile in different dose groups. The trial was designed to primarily assess the safety and tolerability of TG103 in participants with overweight and obesity without diabetes; and weight loss, waist circumference, etc., were assessed as secondary endpoints. The multiple comparison of each dose level of TG103 and placebo was not conducted, so the p-values should be interpreted with consideration. Therefore, the efficacy data were preliminary results. Trials with longer treatment periods are needed to fully assess the efficacy and safety of TG103 and to assess whether its efficacy is sustained. The efficacy of TG103 in participants who are overweight and obese with diabetes also needs to be further investigated.

Given the good safety and potential efficacy of TG103 in participants with overweight and obesity, two phase II trials to further evaluate the efficacy and safety of TG103 in Chinese adults who are overweight or obese without diabetes (NCT05299697) and with type-2 diabetes (NCT05063253) are ongoing.

## Conclusions

In this trial, all three doses of once-weekly TG103 were well tolerated with an acceptable safety profile. Pharmacokinetic parameters support once-weekly dosing of TG103, and this dosing regimen was associated with preliminary 12-week weight loss without lifestyle interventions. TG103 preliminarily showed great potential for the treatment of participants with overweight and obesity without diabetes. Future trials with larger sample sizes and longer treatment periods are needed to fully assess the efficacy and safety of TG103 in participants who are overweight or obese.

### Supplementary Information

Additional file 1: Table S1. Blood sampling schedule for pharmacokinetic assessments. Table S2. Summary of pharmacokinetic parameters of TG103 after first target dose. Fig. S1. Serum concentration–time curves of TG103 after the first target dose: (A) Linear plots and (B) Semi-logarithmic plots. Error bars represent standard deviation.

## Data Availability

Data used in the present study can be accessed upon request from the corresponding authors.

## Abbreviations

ADA: Anti-drug antibodies
AE: Adverse event
BMI: Body-mass index
CI: Confidence interval
CTCAE: Common Terminology Criteria for Adverse Events
ECG: Electrocardiogram
ECL: Electrochemiluminescence
FAS: Full analysis set
FDA: Food and Drug Administration
GLP-1: Glucagon-like peptide-1
NCI: National Cancer Institute
PD: Pharmacodynamic
PDS: Pharmacodynamics analysis set
PK: Pharmacokinetic
PKCS: Pharmacokinetic concentration analysis set
PKPS: Pharmacokinetic parameter analysis set
QW: Once a week
SAD: Single ascending dose
SAE: Serious adverse event
SC: Subcutaneously
SD: Standard deviation
SE: Standard error
SS: Safety analysis set
RA: Receptor agonist
WHO: World Health Organization
