# Supplementary material for: Safety, tolerability, pharmacokinetics, and pharmacodynamics of TG103 injection in participants who are overweight or obese: a randomized, double-blind, placebo-controlled, multiple-dose phase 1b study
Source: BMC Med. 2024 May 29;22:209. doi: 10.1186/s12916-024-03394-z (PMC11134614; doi:10.1186/s12916-024-03394-z)
Supplement: Supplementary file 1 — Additional file 1: Table S1. Blood sampling schedule for pharmacokinetic assessments. Table S2. Summary of pharmacokinetic parameters of TG103 after first target dose. Fig. S1. Serum concentration–time curves of TG103 after the first target dose: (A) Linear plots and (B) Semi-logarithmic plots. Error bars represent standard deviation. [file 12916_2024_3394_MOESM1_ESM.docx]

**Additional file 1: Table S1.** Blood sampling schedule for pharmacokinetic assessments. **Table S2.** Summary of pharmacokinetic parameters of TG103 after first target dose. **Fig. S1.** Serum concentration-time curves of TG103 after the first target dose: (A) Linear plots and (B) Semi-logarithmic plots. Error bars represent standard deviation.

**Table S1.** Blood sampling schedule for pharmacokinetic assessments

| **Stage** | **Blood sampling schedule** |
| --- | --- |
| 15.0 mg | - Within 2 h before dosing on day 1 and day 8. - 6, 12, 24, 36, 48, 72, 96, 144 and 168 h (before dosing on day 15) after dosing on day 8. - Within 2 h before dosing on day 64, day 71 and day 78. - 6, 12, 24, 36, 48, 72, 96, 144, 168, 336 and 504 h after dosing on day 78. |
| 22.5 mg | - Within 2 h before dosing on day 1, day 8 and day 15. - 6, 12, 24, 36, 48, 72, 96, 144 and 168 h (before dosing on day 22) after dosing on day 15. - Within 2 h before dosing on day 64, day 71 and day 78. - 6, 12, 24, 36, 48, 72, 96, 144, 168, 336 and 504 h after dosing on day 78. |
| 30.0 mg | - Within 2 h before dosing on day 1, day 8, day 15 and day 22. - 6, 12, 24, 36, 48, 72, 96, 144 and 168 h (before dosing on day 29) after dosing on day 22. - Within 2 h before dosing on day 64, day 71 and day 78. - 6, 12, 24, 36, 48, 72, 96, 144, 168, 336 and 504 h after dosing on day 78. |

**Table S2**. Summary of pharmacokinetic parameters of TG103 after first target dose

|  | **15.0 mg**  **(N = 12)** | **22.5 mg**  **(N = 6)** | | **30.0 mg**  **(N = 12)** | |
| --- | --- | --- | --- | --- | --- |
| **T_max_ (h)** | 36.00 (12.07, 96.00) | | 35.89 (35.75, 72.00) | | 48.00 (35.87, 72.13) |
| **C_max_ (ng/mL)** | 469±193 | | 1250±599 | | 1870±596 |
| **AUC_0-t_ (μg*h/mL)** | 56.1±21.4 | | 130±56.0 | | 214±50.9 |
| **AUC_0-inf_ (μg*h /mL)** | 97.0±31.5 | | 194±45.7 | | 305±51.9 |
| **t_1/2_ (h)** | 121±88.2 | | 134±150 | | 87.5±33.2 |
| **V_z_/F (L)** | 26.7±13.6 | | 26.4±35.6 | | 12.9±5.92 |
| **CL/F (L/h)** | 0.17±0.06 | | 0.12±0.03 | | 0.10±0.02 |

Data are described by mean±SD except for T_max_ showed as median (min, max). AUC_0-t_, area under the concentration‐time curve from time zero to time of the last measurable concentration; AUC_0-inf_, area under the concentration‐time curve from time zero to infinity; C_max_, maximum concentration; CL/F, apparent total body clearance; t_1/2_, terminal half-life; T_max_, time to maximum concentration; SD, standard deviation; V_z_/F, apparent total volume of distribution.


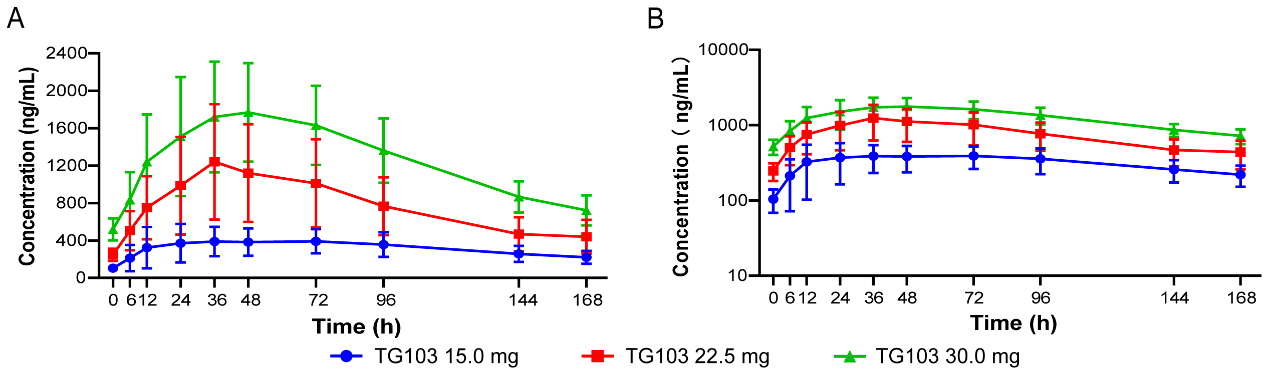


**Fig. S1.** Serum concentration-time curves of TG103 after the first target dose: (A) Linear plots and (B) Semi-logarithmic plots. Error bars represent standard deviation.
